# The role of health literacy within the social determinants of health framework: a cross-sectional study on smoking behavior in Fujian, China

**DOI:** 10.3389/fpubh.2025.1626620

**Published:** 2025-07-23

**Authors:** Zulin Chen, Yikun Zheng, Lihan Lin, Yongjun Chen, Yunting Zheng, Hongmiao Chen

**Affiliations:** ^1^School of Physical Education, Huaqiao University, Quanzhou, China; ^2^Research Center for Sports and Health Sciences, Huaqiao University, Quanzhou, China; ^3^Quanzhou Center for Disease Control and Prevention, Quanzhou, China; ^4^School of Health Management, Fujian Medical University, Fuzhou, China

**Keywords:** tobacco use, smoking behavior, health literacy, social determinants of health/SDoH, educational inequalities, male, cross-sectional study

## Abstract

**Background:**

Smoking is a leading preventable cause of death, and its prevalence varies with social determinants of health (SDoH) such as education, age, and urban/rural residence. Health literacy (HL) may influence tobacco use, but its interplay with SDoH in China is unclear. This study examined associations between HL, key SDoH, and current smoking among residents aged 15–69 in Quanzhou, Fujian, China.

**Methods:**

A cross-sectional survey was conducted in 2024 among 3,200 residents of Quanzhou, Fujian Province, selected via multistage random sampling. Data on smoking status, health literacy, and SDoH were collected using the nationally standardised questionnaire developed by the Chinese Center for Health Education. Associations between smoking status, HL, and SDoH were analyzed using chi-square tests and multivariable logistic regression.

**Results:**

Of 3,200 participants, the overall prevalence of current smoking was 25.680%, with significant gender differences (*p* < 0.001). Given the extremely low prevalence of current smoking among females (0.66%), the following results are based exclusively on male participants. Among males (*n* = 1,533), smoking prevalence was higher in rural areas (51.37%) than in urban areas (41.83%, *p* = 0.002), and increased with age, peaking at 58.74% among those aged 55–64 (*p* < 0.001). Smoking prevalence declined with higher educational attainment, from 54.27% in those with junior high school education to 18.380% in those with a bachelor’s degree or higher (*p* < 0.001). Multivariable logistic regression showed that age was associated with increased odds of smoking (e.g., OR = 5.699, 95% CI: 3.091–10.508 for ages 55–64 vs. 15–24; *p* < 0.001), and higher education was associated with reduced odds (e.g., bachelor’s degree vs. no formal education: OR = 0.180, 95% CI: 0.087–0.374; *p* < 0.001). Among HL dimensions, only inadequate practical health skills remained significantly associated with current smoking (OR = 1.358, 95% CI: 1.015–1.817; *p* = 0.039).

**Conclusion:**

HL and SDoH jointly influenced smoking in Chinese men; low practical health skills and being older, less educated, or from a rural area were linked to higher risk. Strategies that enhance practical health skills and address social disparities may help reduce smoking, supporting Healthy China 2030 and WHO tobacco-control goals.

## 1 Introduction

Tobacco use continues to be the leading preventable cause of death worldwide, with its health burden disproportionately affecting populations along social and economic gradients (1). According to the World Health Organization Global Tobacco Epidemic Report (2021), the prevalence of smoking among males in low- and middle-income countries (36.7%) significantly exceeds that in high-income countries (22.3%) (2). These disparities reflect the systemic inequalities embedded in the social determinants of health (SDoH)—a framework that emphasizes the influence of education, income, geographic location, and access to information on individual health behaviors (3).

As the world’s largest tobacco consumer, China presents complex challenges for tobacco control, with notable variations in smoking prevalence across regions, age groups, and socioeconomic strata (4). In this context, within the broader framework of SDoH, Health literacy (HL) has gained recognition as a modifiable dimension of increasing relevance to public health (5). Defined as the ability to access, understand, evaluate, and apply health information, HL is increasingly recognized as a key determinant of health behavior, including tobacco use (6).

Recent studies have demonstrated that low HL may impair an individual’s understanding of the health risks associated with smoking and reduce their capacity to engage with prevention and cessation strategies (5). Nutbeam’s conceptual model of HL outlines three dimensions—functional, interactive, and critical literacy—that may differentially influence health behaviors depending on context and population (7).

Quanzhou was selected as the research site due to its significant provincial representativeness. Its geographic environment exhibits a strong correspondence with the key characteristics of Fujian Province, encompassing typical landforms such as coastal plains, inland hills, and mountainous regions. This diverse terrain provides a regional reference for examining the impact of environmental factors on health behaviors (8, 9). As the city with the largest resident population in Fujian, Quanzhou’s large population base helps reduce sampling error and enhances the statistical robustness of research findings (10, 11). From an economic perspective, Quanzhou has maintained the highest GDP in the province for more than 20 years. Its diversified industrial structure, led by the private sector, includes key sectors such as manufacturing, cross-border e-commerce, and cultural tourism, which reflect the economic development characteristics of Fujian Province (12). The World Health Organization (WHO) emphasizes the importance of considering internal regional differences in SDoH research (13). Quanzhou, as the cultural core of the Minnan region and a significant diasporic hub, is characterized by a high mobility of its resident population, providing a valuable empirical context for exploring the interactive mechanisms between HL and SDoH (14).

This study addresses this gap by examining the association between smoking status and HL among residents aged 15 to <70 years in Quanzhou, Fujian Province. Using data from a large-scale, population-based survey and applying both descriptive and multivariable statistical analyses, we aim to: (1) describe the current smoking prevalence among residents; (2) assess the independent associations between smoking behavior, HL levels, and key social determinants such as age, education, and residence.

This study aims to explore the associations between HL and the SDoH in relation to smoking behavior in a coastal city of southeastern China. It further seeks to provide evidence-based insights for precision tobacco control strategies—particularly in the context of the “Healthy China 2030” national policy framework.

## 2 Materials and methods

### 2.1 Study population

This cross-sectional study was conducted in Quanzhou City, Fujian Province, China, in 2024. A total of 3,267 residents aged 15 to <70 years who had lived in the city for more than 6 months were initially recruited, of whom 3,200 met the inclusion criteria and were included in the final analysis.

Inclusion criteria were: (1) residency in Quanzhou for at least 6 months at the time of the survey; (2) age between 15 and <70 years; (3) ability to complete the interview independently; and (4) no restriction on household registration.

Exclusion criteria included: individuals residing collectively in institutions such as dormitories, schools, or hospitals, and individuals unable to independently respond to the questionnaire due to mental illness, cognitive impairment, or physical disability.

### 2.2 Study design and procedures

#### 2.2.1 Sampling method

A stratified multi-stage cluster random sampling method was employed in this study, with all random selections conducted using computer-generated random numbers. Between June and October 2024, 40 townships or subdistricts were randomly selected from 13 counties (districts) in Quanzhou, with the sampling within each county (district) being proportionate to population size. In each selected subdistrict, two communities (or villages) were randomly chosen, and within each community, 55 household units were randomly selected. A trained investigator then visited each household and utilized a tablet-based survey application to register eligible household members. One eligible respondent per household was subsequently selected using the Kish method (15).

#### 2.2.2 Data collection

Data were collected using the HL questionnaire developed by the Chinese Center for Health Education, based on the *Health Literacy of Chinese Citizens: Basic Knowledge and Skills* (16). The questionnaire classifies HL into three broad domains: basic knowledge and concepts, healthy lifestyles and behaviors, and basic skills. It can also be categorized into six content-specific literacy domains corresponding to key health issues: scientific health concepts, infectious disease prevention, chronic disease prevention, safety and first aid, basic medical care, and health information. The specific contents of the three domains are as follows: (1) Basic Knowledge and Concepts, comprising 22 items covering topics such as a scientific perspective on health, infectious disease prevention, and chronic disease management; (2) Healthy Lifestyles and Behaviors, comprising 16 items assessing participants’ knowledge of healthy diet, physical exercise, smoking cessation, and moderation of alcohol consumption, as well as their capacity to implement these healthy behaviors; and (3) Basic Skills, comprising 12 items examining whether participants are able to identify, access, and utilize health information when facing health-related issues. The questionnaire has strong internal consistency (Cronbach’s *α* = 0.931) and split-half reliability (Spearman–Brown correlation coefficient = 0.808) in the Chinese context (16).

The questionnaire was digitized and deployed via a mobile application installed on tablet devices, with built-in intelligent prompts to assist data collection. Face-to-face interviews were conducted. Participants completed the electronic questionnaire independently; if a respondent had difficulty reading or writing, trained investigators conducted the interview verbally. All responses were uploaded in real time to a secure data server.

The study was approved by the Ethics Committee of the Quanzhou Center for Disease Control and Prevention (No. 2024003).

#### 2.2.3 Definitions and classification criteria

According to the WHO SDoH framework, structural determinants are defined as the socio-economic and political mechanisms that generate social hierarchies and shape individuals’ access to health opportunities and outcomes (13). In this study, education level, age, and place of residence were selected as key structural SDoH, as they represent principal axes of social stratification and have been consistently associated with health behaviors such as smoking in both international and Chinese contexts (2–4).

The threshold for adequate HL is defined as achieving a total score equal to or greater than 80% of the maximum possible score on the questionnaire, in accordance with the standards set by the National Health Commission of China. Similarly, an individual is considered to possess adequate HL in any of the three HL dimensions or six health issue-specific categories if they achieve a score of 80% or more in the respective dimension, thereby demonstrating adequate HL in that specific dimension.

Smoking was defined as the use of any tobacco products, including electronic cigarettes. Current smoking status was determined based on self-reported behavior in the past 7 days and included both daily and non-daily smokers. The current smoking rate was calculated as the proportion of current smokers among the total sample. Individuals who had smoked in the past but were not currently smoking, along with those who had never smoked, were categorized as non-smokers.

#### 2.2.4 Quality control

All investigators and quality control personnel received standardized training. During data collection, investigators were prohibited from providing any guidance or suggestions to ensure unbiased responses. Quality control officers at the municipal and county levels randomly reviewed 5% of the collected questionnaires by telephone or in-person verification. If more than 20% of the sampled questionnaires from a county were found to be invalid or inconsistent, the entire data collection process in that county was deemed unqualified and repeated.

#### 2.2.5 Statistical analysis

Data were exported from the Fujian Provincial Health Monitoring System Version 1.0, a tablet-based application that required complete questionnaire submission for successful upload, thus ensuring no missing values. Basic data cleaning procedures, including checks for logical consistency and formatting accuracy, were nevertheless performed prior to analysis. Statistical analyses were performed using SPSS Version 26.0. Descriptive statistics were used to summarize the current smoking status. Categorical variables were presented as proportions and compared using chi-square (*χ*^2^) tests. Potential influencing factors of smoking behavior were analyzed using chi-square tests and multivariable logistic regression models. Due to the extremely small number of female smokers, all variables except gender were included in the logistic regression analysis. A two-sided *p*-value of <0.05 was considered statistically significant.

## 3 Result

### 3.1 Basic characteristics of the study population

A total of 3,267 questionnaires were collected, of which 3,200 met the inclusion criteria and were deemed valid after excluding incomplete or ineligible responses, yielding a valid response rate of 97.95%. These 3,200 participants, all aged 15 to <70 years, were included in the final analysis. Of these, 47.91% were male (*n* = 1,533) and 52.09% were female (*n* = 1,667), with a sex ratio of approximately 1:1.08. A majority of the participants resided in rural areas (75.00%, *n* = 2,400), while 25.00% (*n* = 800) lived in urban areas. Age was relatively evenly distributed, with the highest proportion in the 45 to <55-year group (25.97%, *n* = 831), followed by the 55 to <65-year group (24.98%, *n* = 799). Participants aged 15 to <25 years accounted for the smallest group (6.00%, *n* = 192). In terms of education level, the largest proportion had completed junior high or middle school (29.41%, *n* = 941), followed by senior high or vocational school (24.41%, *n* = 781); only 8.19% (*n* = 262) had a bachelor’s degree or higher.

The overall prevalence of current smoking among all participants was 25.68% (*n* = 822). Significant differences in smoking prevalence were observed across demographic subgroups. Males had a substantially higher current smoking rate (49.12%) compared to females (0.66%) (*χ*^2^ = 1031.846, *p* < 0.001). Rural residents reported a higher smoking rate (25.38%) than urban residents (19.38%) (*χ*^2^ = 11.885, *p* = 0.001). Smoking prevalence increased with age, from 7.81% in the 15 to <25-year group to 27.42% in the 65 to <70-year group (*χ*^2^ = 43.278, *p* < 0.001). In terms of educational attainment, participants with junior high/middle school education exhibited the highest smoking rate (33.05%), while those with a bachelor’s degree or above had the lowest (9.54%) (*χ*^2^ = 125.126, *p* < 0.001). All differences were statistically significant (Table 1).

**Table 1:** Demographic characteristics of study participants and smoking prevalence (*n* = 3,200).

Demographic characteristics	Participants, *n* (%)	Current smokers, *n* (%)	Non-smoking, *n* (%)	χ^2^	*p*-value
Gender				1031.846	<0.001
Male	1,533 (47.91)	753 (49.12)	780 (50.88)		
Female	1,667 (52.09)	11 (0.66)	1,656 (99.34)		
Residence				11.885	0.001
Urban	800 (25.00)	155 (19.38)	645 (80.63)		
Rural	2,400 (75.00)	609 (25.38)	1791 (74.63)		
Age group				43.278	<0.001
15–<25	192 (6.00)	15 (7.81)	177 (92.19)		
25–<35	334 (10.44)	73 (21.86)	261 (78.14)		
35–<45	734 (22.94)	151 (20.57)	583 (79.43)		
45–<55	831 (25.98)	224 (26.96)	607 (73.04)		
55–<65	799 (24.98)	216 (27.03)	583 (72.97)		
65–<70	310 (9.69)	85 (27.42)	225 (72.58)		
Education level				125.126	<0.001
Illiterate or low literacy	407 (12.72)	41 (10.07)	366 (89.93)		
Primary School	845 (26.41)	226 (26.75)	619 (73.25)		
Junior high school/middle school	941 (29.41)	311 (33.05)	630 (66.95)		
Senior high school/vocational high school/technical secondary school	466 (14.56)	111 (23.82)	355 (76.18)		
College diploma/associate degree	276 (8.72)	50 (17.92)	229 (82.08)		
Bachelor’s degree or higher	262 (8.19)	25 (9.54)	237 (90.46)		

### 3.2 Current smoking status among males by sociodemographic characteristics

Given that only 11 female participants reported current smoking behavior, accounting for a prevalence of merely 0.66%, further analysis of smoking-related factors was limited to male participants.

Among the 1,533 male respondents, the overall prevalence of current smoking was 49.12%. Significant differences in smoking rates were observed across subgroups defined by residence, age, and education level.

Males residing in rural areas demonstrated a significantly higher smoking prevalence (51.37%) compared to those in urban areas (41.83%) (*χ*^2^ = 10.044, *p* = 0.002). With regard to age, smoking prevalence increased with age up to 65 years and then slightly declined. The highest smoking rate was observed in the 55 to <65-year group (58.74%), followed by the 45 to <55-year group (54.31%), while the lowest was found in the 15 to <25-year group (13.27%) (*χ*^2^ = 79.985, *p* < 0.001). Education level was also significantly associated with smoking behavior (*χ*^2^ = 104.834, *p* < 0.001). The highest prevalence was observed among males with only a junior high/middle school education (54.27%) and those with a senior high/vocational/technical education (40.59%). In contrast, participants with a bachelor’s degree or higher had the lowest prevalence (18.38%).

These findings suggest that lower educational attainment, older age, and rural residency are associated with increased likelihood of current smoking among men (Table 2).

**Table 2:** Sociodemographic profile and current smoking status of male participants (*n* = 1,533).

Demographic characteristics	Current smokers, *n* (%)	Non-smoking, *n* (%)	χ^2^	*p*-value
Residence			10.044	0.002
Urban	151 (41.83)	210 (58.17)		
Rural	602 (51.37)	570 (48.63)		
Age group			79.985	<0.001
15–<25	15 (13.27)	98 (86.73)		
25–<35	72 (45.00)	88 (55.00)		
35–<45	150 (44.91)	184 (55.09)		
45–<55	219 (53.41)	191 (46.59)		
55–<65	215 (58.74)	151 (41.26)		
65–<70	82 (54.67)	68 (45.33)		
Education level			104.834	<0.001
Illiterate or low literacy	40 (59.70)	27 (40.30)		
Primary school	223 (62.64)	133 (37.36)		
Junior high school/middle school	305 (54.27)	257 (45.73)		
Senior high school/vocational high school/technical secondary school	110 (40.59)	161 (59.41)		
College diploma/associate degree	50 (35.46)	91 (64.54)		
Bachelor’s degree or higher	25 (18.38)	111 (81.62)		

### 3.3 Univariate analysis of the association between HL and current smoking among males

Chi-square tests were performed to examine the univariate associations between HL and current smoking status among male participants (Table 3). The results indicated significant differences in smoking prevalence across several HL domains.

**Table 3:** Univariate analysis of health literacy and current smoking among males (*n* = 1,533).

Domains	Current smoking, *n* (%)	Non-smoking, *n* (%)	χ^2^	*p*-value
Health literacy			9.755	0.002
Adequate	226 (43.55)	293 (56.45)		
Inadequate	527 (51.97)	487 (48.03)		
Basic knowledge & concepts			14.862	<0.001
Adequate	316 (43.89)	404 (56.11)		
Inadequate	437 (53.75)	376 (46.25)		
Lifestyle & behaviors			3.110	0.078
Adequate	227 (45.86)	268 (54.14)		
Inadequate	526 (50.67)	512 (49.33)		
Practical skills			1.497	0.221
Adequate	247 (46.96)	279 (53.04)		
Inadequate	506 (50.25)	501 (49.75)		

Participants with adequate overall HL had a significantly lower prevalence of current smoking (43.55%) compared to those with inadequate HL (51.97%) (*χ*^2^ = 9.755, *p* = 0.002). A similarly significant association was found in the domain of basic health knowledge and concepts, where the current smoking rate was 43.89% among those with adequate literacy versus 53.75% among those with inadequate literacy (*χ*^2^ = 14.862, *p* < 0.001).

In contrast, although smoking prevalence differed slightly across the domains of healthy lifestyle and behaviors (45.86% vs. 50.67%) and practical health skills (46.96% vs. 50.25%), these differences were not statistically significant (*p* = 0.078 and *p* = 0.221, respectively).

### 3.4 Multivariable logistic regression analysis of factors associated with male smoking behavior

A multivariable logistic regression analysis was conducted to identify independent factors associated with current smoking among male participants (Table 4). The model included variables related to residence, age group, education level, HL, basic health knowledge, lifestyle & behaviors and practical skills. Variable coding and assignment strategies are detailed in Table 5.

**Table 4:** Multivariable logistic regression analysis of factors associated with male smoking behavior.

Variable	*β*	S. E.	Wald *χ*^2^	*p*-value	OR	95%CI
Residence						
Urban*	–	–	–	–	–	–
Rural	0.077	0.138	0.315	0.575	1.081	0.824–1.417
Age Group						
15–<25*	–	–	–	–	–	–
25–<35	1.751	0.328	28.499	<0.001	5.763	3.029–10.961
35–<45	1.571	0.307	26.169	<0.001	4.810	2.635–8.779
45–<55	1.655	0.308	28.942	<0.001	5.232	2.863–9.561
55–<65	1.740	0.312	31.073	<0.001	5.699	3.091–10.508
65–<70	1.533	0.343	19.958	<0.001	4.632	2.364–9.076
Education level						
Illiterate or low literacy*	–	–	–	–	–	–
Primary school	0.130	0.275	0.223	0.636	1.139	0.665–1.951
Junior high school/middle school	−0.153	0.276	0.309	0.579	0.858	0.499–1.474
Senior high school/vocational high school/technical secondary school	−0.500	0.300	2.781	0.095	0.607	0.337–1.092
College diploma/associate degree	−0.830	0.339	5.979	0.014	0.436	0.224–0.848
Bachelor’s degree or higher	−1.716	0.373	21.132	<0.001	0.180	0.087–0.374
Health literacy						
Adequate*	–	–	–	–	–	–
Inadequate	−0.087	0.217	0.161	0.688	0.916	0.599–1.403
Basic knowledge & concepts						
Adequate*	–	–	–	–	–	–
Inadequate	−0.214	0.156	1.886	0.170	0.807	0.595–1.096
Lifestyle & behaviors						
Adequate*	–	–	–	–	–	–
Inadequate	0.112	0.167	0.449	0.503	1.119	0.806–1.552
Practical skills						
Adequate*	–	–	–	–	–	–
Inadequate	0.306	0.149	4.242	0.039	1.358	1.015–1.817

*indicates the reference group in the logistic regression analysis.

**Table 5:** Coding scheme for logistic regression variables.

Code	Variable	Value assignment
Y	Smoking status	1 = Current Smoker; 0 = Never Smoker
X_1_	Residence type	1 = Urban; 2 = Rural
X_2_	Age group (years)	1 = Illiterate or Low Literacy; 2 = Primary School; 3 = Junior High School/Middle School; 4 = Senior High School/Vocational High School/Technical Secondary School; 5 = College Diploma/Associate Degree; 6 = Bachelor’s Degree or Higher
X_3_	Education level	1 = 15–<25; 2 = 25–<35; 3 = 35–<45; 4 = 45–<55; 5 = 55–<65; 6 = 65–<70
X_4_	Health literacy	0 = Inadequate; 1 = Adequate
X_5_	Basic knowledge & concepts	0 = Inadequate; 1 = Adequate
X_6_	Lifestyle & behaviors	0 = Inadequate; 1 = Adequate
X_7_	Practical skills	0 = Inadequate; 1 = Adequate

Based on the multivariable logistic regression analysis, several factors were identified as significantly associated with male smoking behavior. Age group emerged as a significant predictor, with older age groups demonstrating progressively higher odds of being current smokers compared to the reference group (15–<25 years). Specifically, the odds of smoking were 5.76 times higher for those aged 25–<35 years (OR = 5.763, 95% CI: 3.029–10.961, *p* < 0.001), 4.81 times higher for those aged 35–<45 years (OR = 4.810, 95% CI: 2.635–8.779, *p* < 0.001), 5.23 times higher for those aged 45–<55 years (OR = 5.232, 95% CI: 2.863–9.561, *p* < 0.001), 5.70 times higher for those aged 55–<65 years (OR = 5.699, 95% CI: 3.091–10.508, *p* < 0.001), and 4.63 times higher for those aged 65–<70 years (OR = 4.632, 95% CI: 2.364–9.076, *p* < 0.001). Education level was also significantly associated with smoking behavior. Participants with a Bachelor’s Degree or higher had 82% lower odds of smoking (OR = 0.180, 95% CI: 0.087–0.374, *p* < 0.001), while those with a College Diploma/Associate Degree had 56% lower odds (OR = 0.436, 95% CI: 0.224–0.848, *p* = 0.014).

In contrast, education levels such as primary school, junior high school, and senior/vocational high school did not show statistically significant associations with smoking behavior compared to the reference group of illiterate or low literacy. Practical skills were also found to significantly affect smoking behavior, with participants having inadequate practical skills showing 1.36 times higher odds of smoking (OR = 1.358, 95% CI: 1.015–1.817, *p* = 0.039). On the other hand, residence (urban vs. rural), HL (adequate vs. inadequate), basic health knowledge and concepts (adequate vs. inadequate), and lifestyle behaviors (adequate vs. inadequate) did not show statistically significant associations with smoking behavior. Specifically, residence had an OR of 1.081 (95% CI: 0.824–1.417, *p* = 0.575), HL showed an OR of 0.916 (95% CI: 0.599–1.403, *p* = 0.688), basic health knowledge and concepts had an OR of 0.807 (95% CI: 0.595–1.096, *p* = 0.170), and lifestyle behaviors had an OR of 1.119 (95% CI: 0.806–1.552, *p* = 0.503), all of which were not statistically significant. It is important to note that the association between HL and smoking behavior became non-significant after adjusting for variables such as age and education level. These findings highlight the strong association of age, education level, and practical skills with male smoking behavior, while other factors did not show significant associations.

## 4 Discussion

This cross-sectional study examined the prevalence of smoking among residents aged 15–70 years in Quanzhou, a coastal city in southeastern China, and explored its associations with various SDoH, including HL as a key modifiable component. Owing to the negligible prevalence of smoking among female participants, the analyses in this study are predominantly based on male data. Specifically, the overall smoking prevalence was 25.68%, with a remarkably low rate of only 0.66% among females, indicating a substantial gender-based disparity in smoking behaviors. This finding is consistent with the results reported by Xia et al. (17), and may be attributed to the social norms embedded in traditional Chinese culture that discourage smoking among women (18–20). These observations suggest that gender should be considered in tobacco control policies, and that future qualitative studies are needed to further explore the sociocultural mechanisms underlying the extremely low smoking prevalence among women in China (21).

In relation to SDoH, univariate analysis showed that smoking prevalence was significantly higher among rural residents than urban residents, which may reflect disparities in health education, access to cessation resources, or cultural norms that normalize smoking in rural contexts (22). Smoking rates increased with age, peaking in the 55–<65 age group, potentially due to cohort effects, prolonged exposure to smoking environments, and lower cessation success among older adults (23). In addition, multivariable logistic regression analysis revealed that educational attainment was inversely associated with smoking; individuals with only junior high school education had the highest prevalence, while those with a bachelor’s degree or higher had significantly lower odds of current smoking, with a risk ratio of only 0.187 compared to illiterate or semi-literate individuals. This trend is in agreement with prior studies by Jin et al. (24) and Deng et al. (25), both of which identified education as a key predictor of smoking behavior. These findings indicate the importance of prioritizing rural, lower-educated, and older populations in targeted tobacco control interventions.

Multivariable logistic regression analysis further confirmed the significant effects of age on smoking behavior among men. Male participants aged 25–<35 had 5.763 times the odds of smoking compared to those aged 15–<25. This age group may be particularly vulnerable due to being in a critical stage of career development, facing elevated work stress, more frequent social interactions, and greater financial independence, all of which may facilitate the use of nicotine as a coping mechanism (26). Furthermore, this age group is more susceptible to peer influence, and in certain occupations, workplace culture may reinforce smoking behavior (27). These findings suggest the necessity of designing context-specific tobacco control strategies that address occupational and psychosocial risk environments.

Regarding HL, univariate analysis revealed that male participants with adequate HL—specifically in the domains of Basic Knowledge & Concepts, Lifestyle & Behaviors, and Practical Skills—had lower smoking prevalence compared to those with inadequate HL. This is consistent with findings from Li et al. (28) and Guo et al. (29), who reported that higher HL levels are associated with healthier decision-making behaviors. However, after adjusting for sociodemographic variables such as age and education, multivariable logistic regression showed that overall HL, as well as the Basic Knowledge & Concepts and Lifestyle & Behaviors dimensions, were not significantly associated with smoking behavior. This suggests that the influence of these aspects of HL diminishes when other structural factors, such as age and education, are taken into account (30). These findings highlight the need to consider HL in conjunction with other SDoH when developing public health interventions.

In addition, These findings align with the knowledge-action gap, a well-established concept in public health, wherein possessing health knowledge does not necessarily translate into behavior change, particularly in addictive behaviors like smoking (6, 31). In the context of smoking, while individuals may be aware of the risks, psychological dependence, addiction, and social norms are significant barriers to adopting healthier behaviors (6, 32). Furthermore, the findings suggest that smoking behavior is strongly influenced by cultural factors and social influences, which often override individual health knowledge (33). The lack of significance for Basic Knowledge & Concepts and Lifestyle & Behaviors may be explained by the cultural normalcy of smoking in certain environments, particularly in rural settings, where smoking may be ingrained as part of the social fabric (34). In such contexts, individuals may continue smoking despite knowing its health risks, as the behavior may be normalized in their social and work environments, thus reducing the impact of basic health knowledge (27). In addition, smoking is often a coping mechanism for stress and emotional issues, further complicating the role of knowledge in smoking cessation (35, 36).

Practical Skills, which encompass the ability to apply health knowledge effectively in real-life situations, were significantly associated with smoking behavior. This suggests that self-efficacy, the belief in one’s ability to make decisions and take actions that influence health outcomes, is a key determinant in smoking cessation (37). Individuals with better practical skills are more likely to resist smoking triggers and adopt effective cessation strategies, underscoring the importance of not just providing health information but also equipping individuals with the skills to act on that information (38). This aligns with findings from other studies, which suggest that skills-based interventions, such as coping strategies for managing cravings and stress, can significantly improve smoking cessation rates (39, 40).

Additionally, the findings suggest that while HL in its functional and interactive forms may have a limited impact, Critical HL, as conceptualized by Nutbeam in 2000, may be more relevant for influencing smoking behavior (7). Critical HL, which involves the ability to critically analyze health information and understand the social and environmental factors influencing health decisions, could provide individuals with the tools to resist social pressures and make informed, autonomous decisions about smoking cessation (41, 42). Critical HL may enable individuals to question societal norms, including the normalization of smoking, and to adopt behaviors that align with their long-term health goals (28, 41). As such, interventions aimed at enhancing Critical HL could be more effective than those focused solely on increasing functional health knowledge, as they equip individuals to engage with health information in a more critical, contextual manner, ultimately leading to more sustained behavior change (41–43). Accordingly, future tobacco control efforts should not rely solely on knowledge dissemination, but should emphasize the development of critical HL competencies, including risk interpretation, structural awareness, and participatory decision-making, in order to enhance behavior change outcomes.

This study is subject to several limitations that constrain the interpretation and generalizability of the findings. First, the exclusive focus on Quanzhou limits the ability to extrapolate the results to regions with divergent developmental contexts, such as inland or western China. Additionally, the cross-sectional design precludes causal inference regarding the relationships between HL, SDoH, and smoking behavior. Critically, the underrepresentation of key demographic strata impedes comprehensive analysis: the exclusion of adults aged ≥70 years limits the life-course perspective, and the extreme scarcity of female smokers (*n* = 11, 0.66% of the sample) fundamentally precludes gender-stratified exploration, limiting the study’s ability to further analyze female smoking behavior and its associated factors, while also indicating that the conclusions are primarily applicable to male populations. Furthermore, the exclusive reliance on self-reported data introduces dual measurement biases, including probable underreporting of smoking behavior due to social desirability pressures and potential overestimation of HL levels due to recall inaccuracies. Although core sociodemographic confounders were adjusted for in the analyses, several key structural SDoH were not included. Due to data sensitivity and the design limitations of the standardized questionnaire, data on critical upstream SDoH such as economic status (e.g., household income), occupational exposures (e.g., job strain), social capital (e.g., support networks), and exposure to tobacco control policies were not collected, as these domains were not explicitly incorporated into the survey instrument. As a result, the structural dimension of SDoH may not have been accurately captured, potentially introducing bias in the estimated effects of education, age, and residence, and limiting the ability to reflect broader macro-level influences on smoking behavior. Future research should address these constraints by employing longitudinal designs to establish temporal precedence, purposive sampling of vulnerable subgroups for a more comprehensive analysis, and integrating biochemical verification alongside self-reports to mitigate biases. Additionally, multilevel modeling incorporating community-level SDoH indicators is needed to account for the broader contextual factors influencing smoking behavior. These steps will help refine our understanding of the complex relationships between HL, SDoH, and smoking, and improve the generalizability and accuracy of future findings.

## 5 Conclusion

This study highlights a significant prevalence of smoking among residents in Quanzhou, with HL identified as a critical and modifiable factor within the broader framework of SDoH. Smoking prevalence is notably associated with factors including geographic location, age, and education, especially affecting rural populations, young men, and individuals with lower educational attainment. To effectively address smoking in these high-risk groups, interventions should prioritize HL enhancement through tailored educational strategies. For rural communities, accessible smoking cessation support via township health centers and mobile health technologies can address barriers like limited healthcare access. Among young men, early educational initiatives in schools and workplaces should emphasize the risks of smoking and benefits of cessation, employing targeted communication reflecting their social contexts. For those with lower educational levels, simplified visual aids and interactive health information are essential to bridge HL gaps effectively. By integrating HL improvements with culturally sensitive and context-specific approaches, these recommendations align with the goals of Healthy China 2030 and global tobacco control initiatives outlined by WHO.

## Data Availability

The original contributions presented in the study are included in the article/Supplementary material, further inquiries can be directed to the corresponding author.
